# Supplementary material for: Telomere Dysfunction in Pediatric Patients with Differences/Disorders of Sexual Development
Source: Biomedicines. 2024 Mar 2;12(3):565. doi: 10.3390/biomedicines12030565 (PMC10968223; doi:10.3390/biomedicines12030565)
Supplement: Supplementary file 1 [file biomedicines-12-00565-s001.zip › biomedicines-2818047-supplementary.pdf]

Supplementary Table S1: Clinical and cytogenetic characteristics of the DSD patient cohort

Identifiers	Civilian Sexes	Ages in months	Reason for consultation	FISH (SRY)	Karyotypes	Additional chromosome aberrations
YH001	M	0,00	DSD	XY (*SRY*+)	46,XY	
YH002	M	178,00	DSD (micro penis)	XY (*SRY*+)	46,XY	
YH003	F	3,00	Prader DSD Type I	XX (*SRY*-)	46,XX	
YH004	M	0,00	DSD	XY (*SRY*+)	46, XY	⇒ DNA Fragmentation<br>⇒ Dicentric with interstitial telomeres
YH005	M	4,00	DSD	XX (*SRY*-)	46,XX	
YH006	M	33,00	DSD	XY (*SRY*+)	46,XY	
YH007	M	19,00	DSD (Hypospadias)	XY (*SRY*+)	46,XY	
YH008	M	120,00	DSD	28% XY/ 57% X (*SRY*+)	46,XY/ 45X	
YH009	M	7,00	DSD	57,5% XY/ 42,5% XXY (*SRY*+)	46,XY/47,XXY	
YH010	F	42,00	DSD	XX (*SRY*-)	46,XX	
YH011	M	1,00	DSD	XY (*SRY*+)	46,XY	
YH012	M	166,00	DSD	XY (*SRY*+)	46,XY	
YH013	F	3,00	DSD	XY (*SRY*+)	46,XY	
YH014	F	0,00	DSD	XX (*SRY*-)	46,XX	⇒ Chromosomal breakage<br>⇒ Loss of a long arm of chromosome 15
YH015	F	180,00	DSD	71% XX /29% X(*SRY*-)	46,XX/45,X	
YH016			DSD	XX (*SRY*-)	46,XX	⇒ Fusion between chromatids of chromosomes (8 and 10)
YH017	M	13,00	DSD	XY (*SRY*+)	46,XY	⇒ Fusion between chromatids of chromosomes (15 and 4)<br>⇒ Fusion between chromatids of chromosomes (15 and 9)
YH018	ND	0,00	DSD	XY (*SRY*+)	46,XY	
YH019	ND	0,00	DSD	XX (*SRY*-)	46,XX	
YH020	M	130,00	Hypospadias post DSD	XY (*SRY*+)	46,XY	⇒ Chromosomal breakage
YH021	ND	0,00	DSD	XY (*SRY*+)	46,XY	
YH022	M	27,00	Micro penis	XY (*SRY*+)	46,XY	
YH023	F	23,00	Congenital genital malformation	XX (*SRY*-)	46,XX	
YH024	M	11,00	DSD	XY (*SRY*+)	46,XY	
YH025	F	17,00	External Genitalia Anomaly	XX (*SRY*-)	46,XX	
YH026	F	0,00	DSD	XX (*SRY*-)	46,XX	
YH027	F	0,00	DSD	XX (*SRY*-)	46,XX	
YH028	ND	0,00	DSD + polymalformation	XY (*SRY*+)	46,XY	
YH029	M	1,00	DSD	XY (*SRY*+)	46,XY	
YH030	M	7,00	DSD	XX (*SRY*-)	46,XX	⇒ Acentric chromosome
YH031	F	30,00	Anomalies des OGE	XY (*SRY*+)	46,XY	
YH032	M	1,00	DSD	XY (*SRY*+)	46,XY	⇒ Chromosomal breakage
YH033	F	35,00	DSD	XX (*SRY*-)	46,XX	⇒ DNA Fragmentation
YH034	F	3,00	DSD	XX (*SRY*-)	46,XX	
YH035	F	2,00	DSD	XY (*SRY*+)	46,XY	
